# Epidemiological Profile and Quality Indicators in Patients with Acute Coronary Syndrome in Northern Minas Gerais - Minas Telecardio 2 Project

**DOI:** 10.5935/abc.20160095

**Published:** 2016-08

**Authors:** Bárbara Campos Abreu Marino, Milena Soriano Marcolino, Rasível dos Santos Reis Júnior, Ana Luiza Nunes França, Priscilla Fortes de Oliveira Passos, Thais Ribeiro Lemos, Izabella de Oliveira Antunes, Camila Gonçalves Ferreira, André Pires Antunes, Antonio Luiz Pinho Ribeiro

**Affiliations:** 1 Centro de Telessaúde do Hospital das Clínicas da Universidade Federal de Minas Gerais Rede de Teleassistência de Minas Gerais, Minas Gerais, MG – Brasil; 2 Faculdade de Medicina da Universidade Federal de Minas Gerais, Minas Gerais, MG – Brasil; 3 Secretaria de Estado de Saúde do Governo de Minas Gerais, Minas Gerais, MG – Brazil; 4 Universidade Estadual de Montes Claros, Minas Gerais, MG – Brasil; 5 Serviço de Cardiologia e Cirurgia Cardiovascular; Hospital das Clínicas da Universidade Federal de Minas Gerais, Belo Horizonte, Minas Gerais, MG – Brazil

**Keywords:** Acute Coronary Syndrome / epidemiology, Health Profile, Quality Indicators, Health Care, Telemedicine

## Abstract

**Background:**

Coronary artery disease is the main cause of death in Brazil. In the Brazilian public health system, the in-hospital mortality associated with acute myocardial infarction is high. The Minas Telecardio 2 Project (Projeto Minas Telecardio 2) aims at implementing a myocardial infarction system of care in the Northern Region of Minas Gerais (MG) to decrease hospital morbidity and mortality. The aim of this study was to describe the profile of the patients with acute coronary syndrome (ACS) cared for in the period that preceded the implementation of the system of care.

**Methods:**

Observational, prospective study of patients with ACS admitted between June 2013 and March 2014 to six emergency departments in Montes Claros, MG, and followed up until hospital discharge.

**Results:**

During the study period, 593 patients were admitted with a diagnosis of ACS (mean age 63 ± 12 years, 67.6% men), including 306 (51.6%) cases of unstable angina, 214 (36.0%) of ST-elevation myocardial infarction (STEMI), and 73 (12.3%) of non-ST-elevation myocardial infarction (NSTEMI). The total STEMI mortality was 21%, and the in-hospital mortality was 17.2%. In the STEMI patients, 46,0% underwent reperfusion therapy, including primary angioplasty in 88 and thrombolysis in six. Overall, aspirin was administered to 95.1% of the patients within 24 hours and to 93.5% at discharge, a P2Y_12_ inhibitor was administered to 88.7% participants within 24 hours and to 75.1% at discharge. A total of 73.1% patients received heparin within 24 hours.

**Conclusion:**

We observed a low reperfusion rate in patients with STEMI and limited adherence to the recommended ACS treatment in the Northern Region of MG. These observations enable opportunities to improve health care.

## Introduction

Myocardial infarction (MI) is the main cause of death in Brazil (8.8% in 2012)^1^ and worldwide.^2^ In Brazil, the mortality of patients with MI is higher in the public health system when compared with the mortality observed in the private system.^3,4^ This occurs because MI patients cared for in the public health system face challenges to have access to intensive care units, methods of reperfusion, and effective therapeutic measures established for MI.^3^

Data from several registries have shown that reperfusion therapy is deficient in many countries, even when available to patients with no contraindication for the procedure.^5^ In patients with ST-elevation MI (STEMI), lack of reperfusion therapy is an independent predictor of mortality. Also, a delay in starting reperfusion therapy in patients who have access to this treatment may influence their outcomes.^6^

**Figure 1:**
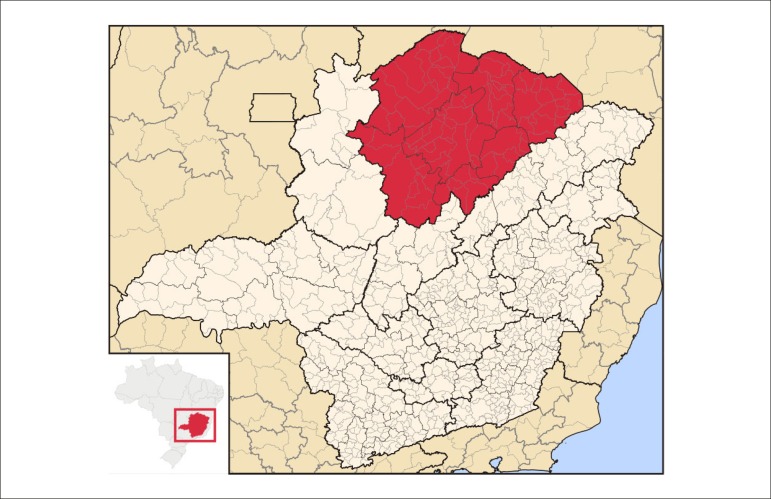
Geographical location of the northern of Minas Gerais. Source: Abreu, RL.

The Brazilian Ministry of Health has established a priority for the MI system of care,^7^ but the national experience in this area is still lacking.^8-12^ The Northern Region of Minas Gerais (MG) is a geographical area with adverse and peculiar conditions, such as low socioeconomic level, large territorial extension (which covers 89 municipalities), and logistical challenges that affect patient care, including unpaved roads, routes that require transportation by ferry boats, and ambulance interception^13,14^ (Figure 1). The implementation of an MI system of care in such conditions is particularly challenging.

The aim of this study was to describe the clinical and epidemiological profile of patients with acute coronary syndrome (ACS) cared for in the Northern Region of MG, with emphasis on quality indicators and evaluation of outcomes in individuals treated in the hospital emergency system who participated in the Minas Telecardio 2 Project (*Projeto Minas Telecardio 2*).

## Methods

### The Minas Telecardio 2 Project

This research project was developed by the Telehealth Network of Minas Gerais (*Rede de Teleassistência de Minas Gerais*), a partnership of six public universities in MG, coordinated by the University Hospital at the *Universidade Federal de Minas Gerais*. The project consisted in implementing an MI system of care in the Northern Region of MG, according to the Directive Number 2994 of December 2011 by the Brazilian Ministry of Health.^7^ The design of this study was quasi-experimental, conducted in three phases: establishment of a baseline, implementation of the MI system of care, and re-evaluation of indicators after implementation. All phases are complete.

The first phase, analyzed in this study,consisted of a prospective cohort of all cases of confirmed ACS diagnoses that presented via the Brazilian public health system (*Sistema Único de Saúde*, SUS) from June 19, 2013, to March 31, 2014, and that were admitted at one of the six Montes Claros emergency departments, where patients with ACS were routinely referred to in the region.

### Structure of myocardial infarction care in the Extended Northern Region of Minas Gerais

**Figure 2:**
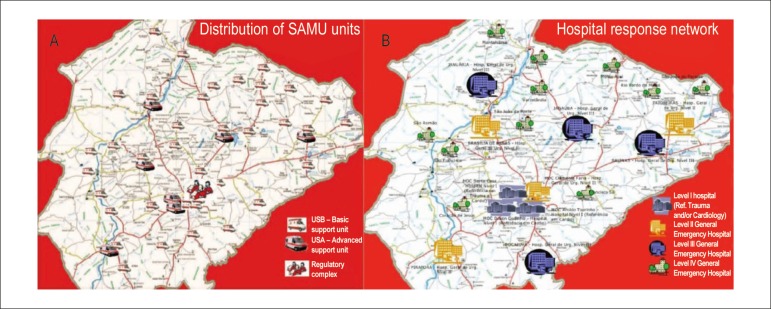
Prehospital and hospital structures in the Expanded Northern Region of Minas Gerais. A: Distribution of SAMU units, B: Network hospital response. Level I hospitals with catheterization rooms in Montes Claros. Source: Souza, RF; Santos-Junior, RR. Minas Gerais State Health Department, 2013.

The Northern Region of MG is covered by the prehospital service *(Serviço de Atendimento Móvel de Urgência*, SAMU). This service is organized within the region, distributed in 37 bases with seven advanced ambulances (*unidade de suporte avançado*, USA), 40 basic ambulances (*unidade de suporte básico*, USB) and an intercept vehicle (Figure 2).

Montes Claros is the main city of the region and concentrates the care of MI patients, routinely receives patients referred from other cities. Montes Claros has three high complexity hospitals (*Santa Casa, Dilson Godinho*, and *Aroldo Tourinho*) with four cathlabs, coronary care unit, and cardiovascular surgery, cardiology, and catheterization teams for caring the public health system. In addition to these hospitals, which are considered level I in MI care,^13,15^ Montes Claros also has a level II hospital (*Hospital Universitário Clemente de Faria*), a municipal emergency service (*Hospital Alpheu de Quadros*) with a structure similar to an immediate care unit, and a hospital covered by SUS for elective care, occasionally sought by patients with MI. The hospital network in the area outside Montes Claros comprises 18 regional hospitals (three level II, four level III, and 10 level IV hospitals (Figure 2).

### Data collection

A surveillance system at the SAMU and in the six emergency departments of Montes Claros was established. Using standardized protocols, trained nurses and medical students interviewed the patients admitted to the hospitals and collected their clinical data from medical records. The patients were followed up until hospital discharge. All forms were revised by a specialist (BM).

Among 1016 evaluated patients, 593 had a confirmed diagnosis of ACS and were included in the study. Based on international criteria, these patients were classified as having STEMI, non-ST elevation MI (NSTEMI), or unstable angina (UA).^16-18^

The occurrence of risk factors or comorbidities was recorded, including previous MI (< 90 days = recent, ≥ 90 days = remote), coronary artery bypass graft (CABG) or previous angioplasty, Chagas disease, hypertension (determined from a self-reported diagnosis or use of antihypertensive medication), dyslipidemia, diabetes mellitus, smoking, chronic renal failure requiring dialysis, and family history of coronary artery disease, which was considered positive in patients with a male first-degree relative presenting MI when aged ≤ 55 years or a female first-degree relative presenting MI when aged ≤ 65 years.

We also analyzed the patients' place of provenance and the type of transportation that brought them to the center in Montes Claros.

### Quality measures and outcomes

We evaluated the quality indicators recommended by the guidelines of the American Heart Association (AHA) / American College of Cardiology (ACC):^19,20^

- Implementation and type of reperfusion therapy;
- Medications administered within the first 24 hours and at hospital discharge;
- Time until treatment implementation.

We evaluated the administration of the following medications within 24 hours: aspirin, P2Y_12_ inhibitors (clopidogrel or ticagrelor), heparins (unfractionated or low-molecular-weight), statins, and angiotensin-converting enzyme inhibitors (ACEI) or angiotensin II receptor blockers (ARB). We also evaluated the administration of all the medications above but heparin at hospital discharge.

We analyzed the following time points: prehospital service response time (time between the call and arrival at the location of the service), total duration of prehospital transportation (prehospital service response time + time from the location of care to the hospital in Montes Claros), door-to-ECG time (in patients who underwent ECG in Montes Claros), door-to-balloon time, door-to-needle time, and total ischemia time (time elapsed from pain onset to medical care + door-to-balloon or door-to-needle time). We did not evaluate the door-to-balloon and door-to-needle times, or the total ischemia duration in patients in whom clinical treatment was recommended.

The outcomes evaluated included total mortality (during prehospital and in-hospital care), in-hospital mortality, time from hospitalization to death, major bleeding according to the TIMI classification,^21^ and hemorrhagic stroke.

### Ethical aspects

The study was approved by the Ethics Research Committee of the involved institutions (protocol number 260/09) and was conducted according to the Declaration of Helsinki and Resolution 196/96, which was effective when the study was approved. All patients and representatives of each unit provided informed consent to participate in the study.

### Statistical analysis

Continuous variables are presented as mean ± standard deviation or median (interquartile range), and categorical variables are presented as frequency (%). We used the Kolmogorov-Smirnov test to evaluate the normality of the distribution of continuous variables. Time to treatment in patients with and without an outcome in the STEMI subgroup was compared with Student's *t* test or Mann-Whitney test, depending on their distributions. Two-tailed p values < 0.05 were considered statistically significant. All analyses were performed with the software SPSS version 20.0 (SPSS Inc., Chicago, IL, USA).

## Results

### Patients with acute coronary syndrome

During the study period, 593 patients were admitted with a diagnosis of ACS, including 51.6% with UA, 36% with STEMI, and 12.3% with NSTEMI. The mean age of the patients was 63 ± 12 years and 67.6% were men. Table 1 shows the participants' comorbidities and risk factors, municipality of origin, and type of transportation.

**Table 1:** General and subgroup clinical characteristics of patients with acute coronary syndrome, excluding prehospital deaths (n = 583)

	**All patients (n = 583)**	**STEMI (n = 204)**	**NSTEMI (n = 73)**	**Unstable angina (n = 306)**
Age (years)	63 ± 12	62 ± 13	63 ± 12	63 ± 11
Male gender	138 (67.6)	138 (67.6)	44 (60.3)	165 (53.9)
**Municipality of provenance**				
Montes Claros	250 (42.9)	72 (35.3)	24 (32.9)	154 (50.3)
Other 88 municipalities	333 (57.1)	132 (64.7)	49 (77.1)	152 (49.4)
**Patient’s provenance**				
Hospital or outpatient clinic in another municipality*	264 (45.3)	117 (57.4)	39 (53.4)	109 (35.6)
Spontaneous demand	229 (39.2)	52 (25.5)	19 (26.0)	159 (52.0)
Prehospital service	49 (8.4)	21 (10.3)	11 (15.1)	16 ( 5.2)
Hospital or outpatient clinic in Montes Claros†	40 (6.9)	14 (6.9)	4 (5.4)	22(7.1)
**Type of transportation‡**				
Own vehicle	229 (39.2)	51 (25.0)	18 (24.7)	155 (50.7)
Prehospital ambulance service	173 (29.7)	100 (49.1)	27 (37.0)	46 (15.0)
Ambulance or municipality health vehicle	166 (28.5)	53 (25.9)	27 (37.0)	86 (28.1)
Municipality bus	15 (2.6)	-	1 (1.4)	19 (6.2)
**Comorbidities and risk factors**				
Hypertension	462 (79.2)	153 (75)	54 (74.0)	255 (83.3)
Dyslipidemia	255 (43.7)	69 (33.8)	31 (42.5)	155 (50.7)
Smoking	116 (19.9)	53 (26.0)	19 (26.0)	44 (14.4)
DM	139 (23.8)	47 (23.0)	18 (24.7)	74 (24.2)
- DM, insulin use	44 (7.5)	13 (6.4)	5 (6.8)	26 (8.6)
Previous use of aspirin	252 (43.2)	58 (28.4)	28 (38.9)	166 (54.2)
Previous stroke	39 (6.7)	11 (5.4)	4 (5.5)	24 (7.8)
Positive family history	235 (40.3)	73 (35.8)	30 (41.1)	132 (43.1)
History of coronary disease	134 (23.0)	24 (11.8)	13 (17.8)	96 (31.4)
- Previous angioplasty	91 (15.6)	20 (9.8)	8 (11.0)	62 (20.3)
- Coronary artery bypass grafting	43 (7.4)	4 (2.0)	5 (6.8)	34 (11.1)
Chagas disease	51 (8.7)	13 (6.4)	9 (12.3)	29 (9.5)
Alcoholism	139 (23.9)	51 (25.0)	15 (20.5)	73 (23.9)
Prior MI	98 (16.8)	33 (16.2)	9 (12.3)	56 (18.3)
- Recent	21 (3.6)	10 (4.9)	2 (2.7)	9 (2.9)
- Remote	77 (13.2)	23 (11.3)	7 (9.6)	47 (15.4)
Angiographic data*				
Location of the culprit lesion				
- Anterior descending	210/511 (41.1)	98/194 (50.5)	24/70 (34.3)	88/247 (35.6)
- Right coronary artery	112/511 (21.9)	67/194 (34.5)	14/70 (20.0)	31/247 (12.6)
- Circumflex	76/511 (14.9)	26/194 (13.4)	21/70 (30.0)	29/247 (11.7)
- Arterial or venous graft	6/511 (1.2)	-	1/70 (1.4)	5/247 (2.0)
- Without significant obstruction (< 50%)	107/511 (20.9)	3/194 (1.5)	10/70 (14.3)	94/247 (38.0)
**Pre-procedure TIMI flow**				
- TIMI 0	150/511 (29.4)	139/194 (71.6)	11/70 (15.7)	-
- TIMI 1	74/511 (14.5)	27/194 (13.9)	15/70 (21.4)	32/247 (13.0)
- TIMI 2	97/511 (19.0)	17/194 (8.8)	17/70 (24.3)	66/247 (26.7)
- TIMI 3	187/511 (36.6)	11/194 (5.7)	27/70 (38.6)	149/247 (60.3)
**Other vessels with lesions ≥ 70% besides the culprit artery**				
- One	116/511 (22.7)	51/194 (26.3)	14/70 (19.7)	51/247 (20.6)
- Two	123/511 (24.0)	64/194 (33.0)	16/70 (22.5)	43/247 (17.4)
- Three	39/511 (7.6)	14/194 (7.2)	8/70 (11.3)	17/247 (6.9)
Angioplasty with stent implantation	250/511 (48.9)	148/194 (76.3)	31/70 (44.3)	71/247 (28.7)
Angiographic post-procedure success†	211/250 (84.4)	114/148 (77.0)	27/31 (90.0)	70/71 (98.6)
**Hospitalization**				
- Length of hospitalization (days)	7 (4-14)	9 (6-16)	10 (7-18)	6 (4-12)
- In-hospital death	55 (9.4)	35 (17.2)	5 (6.8)	15 (4.9)
- Time from hospitalization to death (days)	9 (2-19)	3 (1-15)	(8-30)	19 (8-34)

* Three of the 73 patients with NSTEMI, 59 of the 206 patients with unstable angina, and 10 patients with STEMI did not undergo angiography.

† TIMI 3 flow was considered an angiographic success. STEMI: ST-elevation myocardial infarction; NSTEMI: non-ST-elevation myocardial infarction; DM: diabetes mellitus; MI: myocardial infarction.

A total of 72 patients received exclusively clinical treatment, including 59 with UA, 10 with STEMI, and three with NSTEMI.

Overall, 355 (59.8%) patients underwent revascularization, including angioplasty in 250 and CABG in 105 individuals. Seventeen patients underwent revascularization of the culprit artery which was followed by CABG later on during hospitalization.

Table 2 shows the medications administered within 24 hours and at hospital discharge. Of 181 patients who did not receive beta-blockers within 24 hours, 39 (21.5%) had contraindications for that, including 15 with cardiogenic shock, 12 who were in Killip class 2, and 12 with heart rates (HR) < 60 beats/minute. The remaining 142 (78.5%) patients were in Killip class I or had UA and presented HR > 60 beats/minute.

**Table 2:** Medications administered within 24 hours and at hospital discharge to all patients with acute coronary syndrome and to those in each subgroup, excluding prehospital deaths (n = 583)

**Medication**	**All patients**	**STEMI**	**NSTEMI**	**Unstable angina**
**24 hours**	**n = 583**	**n = 204**	**n = 73**	**n = 306**
Aspirin	563 (96.6)	194 (95.1)	69 (94.5)	300 (98.0)
P2Y_12_ inhibitors	501 (85.9)	181 (88.7)	65 (89.0)	255 (83.3)
Heparins*	372 (63.8)	155 (73.1)	58 (79.5)	160 (52.3)
Beta-blockers	402 (69.0)	139 (68.1)	52 (71.2)	211 (69.0)
Statins	474 (81.3)	168 (82.4)	59 (80.8)	247 (80.7)
ACEI or ARB	391 (67.1)	131 (64.2)	45 (61.6)	215 (70.3)
**Discharge†**	**n = 528**	**n = 169**	**n = 68**	**n = 291**
Aspirin	492 (93.2)	158 (93.5)	64 (94.1)	270 (92.8)
P2Y_12_ inhibitors	362 (68.6)	127 (75.1)	46 (67.6)	183 (62.9)
Beta-blockers	411 (77.8)	136 (80.5)	56 (82.4)	219 (75.5)
Statins	452 (85.6)	149 (88.2)	62 (91.2)	241 (82.8)
ACEI or ARB	337 (63.8)	109 (64.5)	42 (61.8)	186 (63.9)

* Unfractioned heparin or low-molecular-weight heparin. STEMI: ST-elevation myocardial infarction; NSTEMI: non-ST-elevation myocardial infarction; ACEI: angiotensin-converting enzyme inhibitor; ARB: angiotensin receptor blocker II. †Excluding patients who died.

† Excluding patients who died.

Ten patients had hemorrhagic complications, including nine major bleeding episodes according to the TIMI classification^21^ and one hemorrhagic stroke; these patients were discharged without aspirin or a P2Y_12_ inhibitor. The mortality of all patients with ACS was 9.4% and the median time from hospitalization to death was 9 (2-19) days.

### Patients with STEMI

**Figure 3:**
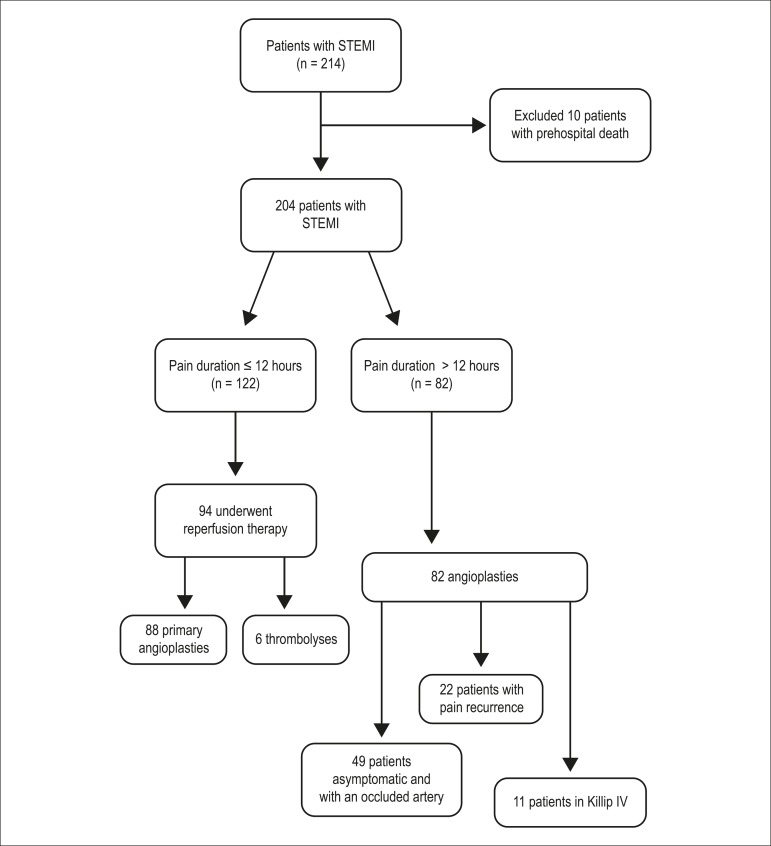
Diagram showing the treatments received by patients with ST-elevation myocardial infarction.

Table 3 shows the time points analyzed in 204 patients with STEMI (excluding those who died before arriving at the hospital). Overall, 57.5% of the patients had pain duration shorter than 12 hours and 46.0% underwent reperfusion therapy, including primary angioplasty in 88 and thrombolysis in six (Figure 3). In the patients elegible for revascularization, reperfusion therapy occurred in 70.6%. Among patients who underwent primary angioplasty, 37.5% had undergone the procedure with a door-to-balloon time greater than 90 minutes. All 82 (42.5%) patients with pain duration greater than 12 hours underwent angioplasty, including 11 patients (5.4% of the total) with cardiogenic shock, 22 (10.8%) with pain recurrence and 49 (24.0%) who presented an occluded artery (TIMI 0), had no pain recurrence, and were asymptomatic.

**Table 3:** Time points assessed in patients with ST elevation myocardial infarction (n = 204)

**Duration (min)**	**All patients (n = 204)**	**In-hospital deaths (n = 35)**	**No deaths (n = 169)**	**p**
Prehospital service response time* (n = 77)	80 (24-177)	112 (40-198)	80 (23-178)	0.79
Transportation time from the place of assistance to the hospital in Montes Claros (n = 77)	45 (18-84)	61 (32-145)	45 (15-71)	0.47
Total duration of prehospital transportation† (n = 77)	177 (50-312)	201 (140-334)	171 (48-304)	0.32
Door-to-ECG time‡ (n = 80)	27 (11-70)	15 (10-31)	30 (11-77)	0.36
Door-to-balloon time (n = 141)	94 (41-386)	90 (31-392)	94 (45-384)	0.62
Door-to-needle time (n = 4)	67 (49-73)	0	67 (49-73)	--
Time between pain onset and request for medical service (n = 204)	486 (248-1657)	414 (215-1521)	549 (249-1521)	0.63
Total ischemia time§ (n =137)	683 (391-1963)	587 (346-2283)	691 (393-1934)	0.91

* Prehospital service response time – amount of time between the call and arrival at the place of service;

† Total prehospital transport time – amount of time for a response from the prehospital service + time from the site of care to a hospital in Montes Claros;

‡ Door-to-ECG time - for patients who underwent ECG in Montes Claros, time between the ECG and the admission;

§ Total ischemia time – time from pain onset until medical care + door-to-balloon or door-to-needle time. In patients who remained in clinical treatment (n = 64), the door-to-balloon time, door-to-needle time, and total ischemia time were not evaluated. The p value refers to the comparison between the groups “in-hospital deaths” versus “no deaths”, analyzed with the Mann-Whitney test.

Regarding antiplatelet drugs in patients with STEMI, 95.1% received aspirin within 24 hours and 93.5% received it upon discharge. A P2Y_12_ inhibitor was administered within 24 hours to 88.7% of the patients and at discharge to 75.1% of them. A total of 73.1% of the patients received heparin, and 68.1% received beta-blockers within 24 hours. Of the 48 (31.9%) patients who did not receive beta blockers, 30 had no contraindications for that and were in Killip class I.

The total number of deaths among the 214 patients with STEMI was 45 (21.0%), including ten (4.6%) that occurred in prehospital care and 35 (17.2%) during hospitalization. The median time from hospitalization to death was 3 (1-15) days.

## Discussion

This study reveals the aspects of ACS treatment away from large Brazilian metropolitan centers, providing information regarding the presentation of the disease and current health care practices in this territory. Our main findings included an increased in-hospital mortality, low reperfusion rates, and excessive treatment with primary angioplasty, even when the transportation time was greater than that recommended by the guidelines, reducing the benefits of primary angioplasty over thrombolysis. In some cases, angioplasty was performed even when the ischemia duration was greater than 12 hours and without symptoms. In addition, we observed a poor adherence to MI quality indicators.

Similar to findings of other ACS registries^22^ and the Brazilian Registry of Acute Coronary Syndrome - (*Registro Brasileiro de Síndrome Coronariana Aguda*, ACCEPT), UA was the most frequent diagnosis (51.7%), followed by STEMI (33.8%), and NSTEMI (12.3%).^23^ However, the prevalence of NSTEMI may have been underestimated, since the troponin assay (cTnI One Step Troponin I Test Device) used for diagnosis in the region is qualitative and has a cutoff value of 0.5 ng/mL. With the use of more sensitive troponin assays, the number of cases of NSTEMI should increase and those of UA should decrease, as described in the literature.^24^

In this study, the in-hospital mortality associated with STEMI was 17.2%, which was above that observed in national and international ACS registries (about 8%).^10,23,25^ However, both these registries - ACCEPT and Global Registry of Acute Coronary Events (GRACE) - included patients cared for in specialized tertiary centers. Of 23 centers participating in the ACCEPT registry, four were private, 15 were philanthropic, and four were public. In addition, all centers had coronary units and catheterization laboratories qualified to perform percutaneous coronary intervention procedures.^23^

The Human Development Index (HDI) of the state of MG is 0.731, but the Northern Region of MG, in contrast with the rest of the state, has an index comparable to that of the poorest states in Brazil.^13^ The municipalities in the region also have important differences in HDIs, ranging from 0.770 in Montes Claros to 0.537 in Bonito de Minas, with an average index of 0.691 in the region.^13^ In 2010, the illiteracy rate in the region was 15.8% and reached 37.2% in some municipalities.^13^

The municipality of Montes Claros functions as a regional municipality polo. More than half of the beds in hospitals covered by SUS are concentrated in small centers with low-technological density, low institutional qualifications, and consequently, precarious resolving capability.^13^ Most roads are still unpaved, and the transportation to the site of medical care in some municipalities requires ferry boats.^13,14^ These particularities impose challenges to the transportation between municipalities.

In order to solve the problem of long distances and lack of ambulances with advanced support, the SAMU teams have organized a system of ambulance interception in which the patients exchange ambulances (from one with basic to another with advanced support, or from one with advanced support to another, also with advanced support, belonging to a different micro region) to transport these patients to locations with more resources to stabilize their conditions and continue their treatments.^14^ However, this maneuver prolongs the transportation time, since it demands time to wait for the ambulance and then to move the patient to another ambulance. This logistical complexity observed in the region delays the implementation of treatment and may have reflected in the high mortality indices observed in our study.

We observed a low (46%) reperfusion rate in patients with STEMI. This reflects the type of treatment that was used in the region, with underutilization of prehospital thrombolysis and centralization of the treatment in Montes Claros, where primary angioplasty was advocated. Considering the large distances in this region and the presence of a regional SAMU, prehospital thrombolysis would improve the reperfusion rates.

Data from the Brazilian ACS registry (ACCEPT*)* have shown a reperfusion rate of 88% in patients with STEMI (85.4% of the cases by primary angioplasty and 14.6% by thrombolysis); however, only 49.6% of the patients in the registry were covered by SUS. In addition, this registry included reference centers and 60.3% of participants from southeastern regions of the country.^26^ In contrast, the GRACE registry has reported a reperfusion rate of 70% and a difference in the type of reperfusion according to the geographic location and type of hospital.^27^ None of these registries included centers with the logistical complexity of the Northern Region of MG.

In the present study, all patients with STEMI and pain duration > 12 hours underwent angioplasty. However, only 40.2% of the patients had a precise indication for the procedure (cardiogenic shock or pain recurrence). The remaining patients were asymptomatic and had occlusion of the culprit artery.

The use of aspirin within 24 hours and at hospital discharge presented satisfactory indices, between 96.6% and 93.2%, in line with similar registries in the literature.^26-29^

Rates of administration of heparin were below those described in other registries: 63.8% in the overall cohort, 73.1% in the subgroup of patients with STEMI, and 52.3% in those with UA. An analysis of the Brazilian data included in the GRACE registry has shown that around 80% of the patients received heparin within 24 hours,^25^ which is aligned with the finding of the ACCEPT registry.^23,26,27^

We observed a high mortality rate and a low reperfusion rate in patients with STEMI, despite the presence in the region of an organized and regional SAMU, three catheterization labs available for intervention procedures, and hospitals with material and human resources. These data show that an improvement in results would require, in addition to material resources, integration of the health care network, implementation and adherence to an evidence-based protocol, and reperfusion methods chosen according to local circumstances. Simple measures of organization of assistance and effective involvement of all of those caring for the patients may promote substantial improvements in care, with potential impact on care indicators, morbidity, and mortality of patients with ACS.^30^ In addition, the implementation of the system of care for ACS requires training and continuous education of the teams, in order to obtain greater adherence to the therapeutic measures established for MI. Finally, implementation of tele-ECG systems in emergency units and ambulances allow, as known, an early diagnosis of STEMI, increasing the possibility of timely reperfusion.^8,31-34^

### Limitations

Due to logistic reasons, we did not collect data from patients with ACS admitted to regional hospitals. However, since the ACS care in the region was centralized in Montes Claros, we believe that the collected data reflect most cases in the region.

## Conclusions

This study observed in the Northern Region of MG a high hospital mortality, low reperfusion rates in patients with STEMI, and insufficient adherence to protocols recommended for the treatment of ACS, suggesting that improvements in the health care process may reduce mortality and improve health care indicators.

## References

[1] Ministério da Saúde Informação em saúde: Estatísticas
vitais.

[2] Naghavi M, Wang H, Lozano R, Davis A, Liang X, Zhou M, GBD 2013 Mortality and Causes of Death Collaborators (2015). Global, regional, and national age-sex specific all-cause and
cause-specific mortality for 240 causes of death, 1990-2013: a systematic
analysis for the Global Burden of Disease Study 2013. Lancet.

[3] Ribeiro AL (2009). The two Brazils and the treatment of acute myocardial
infarction. Arq Bras Cardiol.

[4] Ferreira GM, Correia LC, Reis H, Ferreira CB, Freitas F, Ferreira GM (2009). Increased mortality and morbidity due to acute myocardial
infarction in a public hospital, in Feira de Santana, Bahia. Arq Bras Cardiol.

[5] Kumbhani DJ, Fonarow GC, Cannon CP, Hernandez AF, Peterson ED, Peacock WF, Get With the Guidelines Steering Committee and
Investigators (2013). Predictors of adherence to performance measures in patients with
acute myocardial infarction. Am J Med.

[6] Bassand JP, Danchin N, Filippatos G, Gitt A, Hamm C, Silber S (2005). Implementation of reperfusion therapy in acute myocardial
infarction. A policy statement from the European Society of
Cardiology. Eur Heart J.

[7] Ministério da Saúde (2011). Portaria nº 2.994 de 13 de dezembro de 2011: aprova a linha de cuidado
do infarto agudo do miocárdio e o protocolo de síndromes
coronarianas agudas, cria e altera procedimentos na Tabela de Procedimentos,
Medicamentos, Órteses, Próteses e Materiais Especiais do
SUS.

[8] Marcolino MS, Brant LC, Araujo JG, Nascimento BR, Castro LR, Martins P (2013). Implementation of the myocardial infarction system of care in
city of Belo Horizonte, Brazil. Arq Bras Cardiol.

[9] Solla DJ, Paiva Ide M, Delisle JE, Braga AA, Moura JB, Xd Moraes (2013). Integrated regional networks for ST-segment-elevation myocardial
infarction care in developing countries: the experience of Salvador, Bahia,
Brazil. Circ Cardiovasc Qual Outcomes.

[10] Santos IS, Goulart AC, Brandao RM, Santos RC, Bittencourt MS, Sitnik D (2015). One-year mortality after an acute coronary event and its clinical
predictors: the ERICO study. Arq Bras Cardiol.

[11] Nasi LA, Ferreira-Da-Silva AL, Martins SC, Furtado MV, Almeida AG, Brondani R (2014). Implementation of a dedicated cardiovascular and stroke unit in a
crowded emergency department of a tertiary public hospital in Brazil: effect
on mortality rates. Acad Emerg Med.

[12] Falcao FJ, Alves CM, Barbosa AH, Caixeta A, Sousa JM, Souza JA (2013). Predictors of in-hospital mortality in patients with ST-segment
elevation myocardial infarction undergoing pharmacoinvasive
treatment. Clinics (Sao Paulo).

[13] Torres SF, Belisário SA, Melo EM (2015). A rede de urgência e emergência da
macrorregião Norte de Minas Gerais: um estudo de caso. Saúde Soc São Paulo.

[14] REDE Urgência de Emergência (2011). Manual SAMU Macro Norte.

[15] Marques AJ, Santos RR (2013). As redes de urgência e emergência.

[16] Thygesen K, Alpert JS, Jaffe AS, Simoons ML, Chaitman BR, White HD, Joint ESC/ACCF/AHA/WHF Task Force for the Universal Definition of
Myocardial Infarction (2012). Third universal definition of myocardial
infarction. Circulation.

[17] Steg PG, James SK, Atar D, Badano LP, Blomstrom-Lundqvist C, Borger MA, Task Force on the management of ST-segment elevation acute
myocardial infarction of the European Society of Cardiology (2012). ESC Guidelines for the management of acute myocardial infarction
in patients presenting with ST-segment elevation. Eur Heart J.

[18] Hamm CW, Bassand JP, Agewall S, Bax J, Boersma E, Bueno H, ESC Committee for Practice Guidelines (2011). ESC Guidelines for the management of acute coronary syndromes in
patients presenting without persistent ST-segment elevation: The Task Force
for the management of acute coronary syndromes (ACS) in patients presenting
without persistent ST-segment elevation of the European Society of
Cardiology (ESC). Eur Heart J.

[19] Krumholz HM, Anderson JL, Bachelder BL, Fesmire FM, Fihn SD, Foody JM, American College of Cardiology/American Heart Association Task Force
on Performance Measures, American Academy of Family Physicians, American College of Emergency Physicians, American Association of Cardiovascular and Pulmonary
Rehabilitation, Society for Cardiovascular Angiography and Interventions, Society of Hospital Medicine (2008). ACC/AHA 2008 performance measures for adults with ST-elevation
and non-ST-elevation myocardial infarction: a report of the American College
of Cardiology/American Heart Association Task Force on Performance Measures
(Writing Committee to Develop Performance Measures for ST-Elevation and
Non-ST-Elevation Myocardial Infarction) Developed in Collaboration With the
American Academy of Family Physicians and American College of Emergency
Physicians Endorsed by the American Association of Cardiovascular and
Pulmonary Rehabilitation, Society for Cardiovascular Angiography and
Interventions, and Society of Hospital Medicine. J Am Coll Cardiol.

[20] Cannon CP, Brindis RG, Chaitman BR, Cohen DJ, Cross JT, Drozda JP (2013). 2013 ACCF/AHA key data elements and definitions for measuring the
clinical management and outcomes of patients with acute coronary syndromes
and coronary artery disease: a report of the American College of Cardiology
Foundation/American Heart Association Task Force on clinical data standards
(writing committee to develop acute coronary syndromes and coronary artery
disease clinical data standards). J Am Coll Cardiol.

[21] Serebruany VL, Atar D (2007). Assessment of bleeding events in clinical trials--proposal of a
new classification. Am J Cardiol.

[22] GRACE Investigators (2001). Rationale and design of the GRACE (Global Registry of Acute
Coronary Events) Project: a multinational registry of patients hospitalized
with acute coronary syndromes. Am J Cardiol.

[23] Piegas LS, Avezum A, Guimaraes HP, Muniz AJ, Reis HJ, Santos ES (2013). Acute coronary syndrome behavior: results of a Brazilian
registry. Arq Bras Cardiol.

[24] Giugliano RP, Braunwald E (2014). The year in acute coronary syndrome. J Am Coll Cardiol.

[25] Fox KA, Goodman SG, Klein W, Brieger D, Steg PG, Dabbous O (2002). Management of acute coronary syndromes. Variations in practice
and outcome; findings from the Global Registry of Acute Coronary Events
(GRACE). Eur Heart J.

[26] Piva e Mattos LA, Berwanger O, Santos ES, Reis HJ, Romano ER, Petriz JL (2013). Clinical outcomes at 30 days in the Brazilian Registry of Acute
Coronary Syndromes (ACCEPT). Arq Bras Cardiol.

[27] Eagle KA, Goodman SG, Avezum A, Budaj A, Sullivan CM, Lopez-Sendon J (2002). Practice variation and missed opportunities for reperfusion in
ST-segment-elevation myocardial infarction: findings from the Global
Registry of Acute Coronary Events (GRACE). Lancet.

[28] Eagle KA, Kline-Rogers E, Goodman SG, Gurfinkel EP, Avezum A, Flather MD (2004). Adherence to evidence-based therapies after discharge for acute
coronary syndromes: an ongoing prospective, observational
study. Am J Med.

[29] Wang R, Neuenschwander FC, Lima A, Moreira CM, Santos ES, Reis HJ (2014). Use of evidence-based interventions in acute coronary syndrome -
Subanalysis of the ACCEPT registry. Arq Bras Cardiol.

[30] Berwanger O, Guimaraes HP, Laranjeira LN, Cavalcanti AB, Kodama AA, Zazula AD, Bridge-Acs Investigators (2012). Effect of a multifaceted intervention on use of evidence-based
therapies in patients with acute coronary syndromes in Brazil: the
BRIDGE-ACS randomized trial. JAMA.

[31] Huang RL, Donelli A, Byrd J, Mickiewicz MA, Slovis C, Roumie C (2008). Using quality improvement methods to improve door-to-balloon time
at an academic medical center. J Invasive Cardiol.

[32] Halvorsen S (2012). STEMI treatment in areas remote from primary PCI
centres. EuroIntervention.

[33] Kaifoszova Z, Kala P, Alexander T, Zhang Y, Huo Y, Snyders A (2014). Stent for Life Initiative: leading example in building STEMI
systems of care in emerging countries. EuroIntervention.

[34] Quinn T, Johnsen S, Gale CP, Snooks H, McLean S, Woollard M (2014). Effects of prehospital 12-lead ECG on processes of care and
mortality in acute coronary syndrome: a linked cohort study from the
Myocardial Ischaemia National Audit Project. Heart.

